# *Flavobacterium* sp. strain GJW24 ameliorates drought resistance in *Arabidopsis* and *Brassica*

**DOI:** 10.3389/fpls.2023.1257137

**Published:** 2023-10-13

**Authors:** Hani Kim, Og-Geum Woo, Ji Bin Kim, So-Young Yoon, Jong-Shik Kim, Woo Jun Sul, Jee-Yeon Hwang, Jae-Hoon Lee

**Affiliations:** ^1^ Department of Biology Education, Pusan National University, Busan, Republic of Korea; ^2^ Department of Pharmacology and Neuroscience, Creighton University School of Medicine, Omaha, NE, United States; ^3^ Marine Industry Research Institute for East Sea Rim, Uljin, Republic of Korea; ^4^ Department of Systems Biotechnology, Chung-Ang University, Anseong, Republic of Korea

**Keywords:** GJW24, *Flavobacterium*, plant growth-promoting rhizobacteria, drought tolerance, salt stress tolerance

## Abstract

Candidate strains that contribute to drought resistance in plants have been previously screened using approximately 500 plant growth-promoting rhizobacteria (PGPR) obtained from Gotjawal, South Korea, to further understand PGPR associated with plant drought tolerance. In this study, a selected PGPR candidate, *Flavobacterium* sp. strain GJW24, was employed to enhance plant drought tolerance. GJW24 application to *Arabidopsis* increased its survival rate under drought stress and enhanced stomatal closure. Furthermore, GJW24 promoted *Arabidopsis* survival under salt stress, which is highly associated with drought stress. GJW24 ameliorated the drought/salt tolerance of *Brassica* as well as *Arabidopsis*, indicating that the drought-resistance characteristics of GJW24 could be applied to various plant species. Transcriptome sequencing revealed that GJW24 upregulated a large portion of drought- and drought-related stress-inducible genes in *Arabidopsis*. Moreover, Gene Ontology analysis revealed that GJW24-upregulated genes were highly related to the categories involved in root system architecture and development, which are connected to amelioration of plant drought resistance. The hyper-induction of many drought/salt-responsive genes by GJW24 in *Arabidopsis* and *Brassica* demonstrated that the drought/salt stress tolerance conferred by GJW24 might be achieved, at least in part, through regulating the expression of the corresponding genes. This study suggests that GJW24 can be utilized as a microbial agent to offset the detrimental effects of drought stress in plants.

## Introduction

Various abiotic stresses including heat, cold, UV, drought, high salt, and heavy metals, adversely affect plant productivity (Zhu, 2002). However, plants have developed regulatory mechanisms via plant-microbe interactions as a strategy to overcome these stresses. Plant growth-promoting rhizobacteria (PGPR), which are indigenous to the plant rhizosphere, can play a positive role in plant growth via diverse mechanisms, including enhancement of abiotic stress tolerance (Vejan et al., 2016). When plants face abiotic stresses, PGPR provides plants with adaptation mechanisms such as the regulation of hormones such as indole-3-acetic acid (IAA), cytokinin (CK), gibberellic acid (GA), and abscisic acid (ABA); the production of 1-aminocyclopropane 1-carboxylic acid (ACC) deaminase; the production of antioxidants; the secretion of osmolytes; and the secretion of microbial exopolysaccharide (EPS) (Ma et al., 2020). Although a diverse range of bacteria belonging to *Pseudomonas* and *Bacillus* have been reported as PGPR, the study on PGPR responsible for abiotic stress tolerance in other groups has been relatively poor (Beneduzi et al., 2012).

Drought is one of the abiotic stresses determining plant productivity. It has several adverse effects during plant life cycle, such as the inhibition of photosynthesis, alteration of the respiration rate, disruption of metabolic processes, and improper ion uptake (Hussain et al., 2018). Therefore, to offset these adverse effects, studies on useful PGPR involved in improving plant drought tolerance have been rapidly conducted in recent years. Many studies have revealed that various PGPR ameliorate drought stress resistance in diverse plant species. IAA and GA from several PGPR promote nutrient acquisition and growth of plants, enabling plant survival under drought/salt stress conditions (Ma et al., 2020; Uzma et al., 2022). The treatment of CK-producing *Bacillus subtilis* has been shown to improve drought resistance in *Platycladus orientalis* (Liu et al., 2013). These PGPR-produced phytohormones regulate the formation of root hairs and lateral roots, leading to increased uptake of water and nutrients. PGPR, such as *Bacillus licheniformis* Rt4M10, *Phyllobacterium brassicacearum* STM196, and *Azospirillum brasilense* Sp 245, increase ABA content in plants, consequently ameliorating drought tolerance (Bresson et al., 2013; Cohen et al., 2015). Abiotic stress-induced ethylene inhibits plant growth; however, ACC deaminase (an ACC-degrading enzyme)-producing PGPR can mitigate the adverse effects of abiotic stressors and positively regulate plant growth (Lephatsi et al., 2021). Several reports have demonstrated that ACC deaminase-producing microbes confers enhanced tolerance to drought/salt stress in diverse plant species (Forni et al., 2017). Abiotic stress triggers the production of reactive oxygen species, which are highly toxic to macromolecules and lipids and result in oxidative damage in plant cells (You and Chan, 2015). Some PGPR are involved in regulating the activities of antioxidant enzymes and their gene expression, which mitigates the deleterious effects of abiotic stressors, including drought (Bharti et al., 2016; Batool et al., 2020). Plants utilize compatible osmolytes as osmoprotectants to stabilize macromolecules and membrane structure under drought/salt stress. PGPR-produced EPS is able to help keep the water potential of the rhizosphere high by binding soil particles, thereby ameliorating plant abiotic stress tolerance (Naseem et al., 2018). The existence of PGPR, which produces these osmolytes and EPS, has been widely reported, with a focus on its effect on enhancing resistance to drought/salt stress (Naseem et al., 2018; Khan and Bano, 2019; Ma et al., 2020). Collectively, these studies show that various PGPR strains play crucial roles in ameliorating plant drought stress resistance via diverse mechanisms.

In this research, we show that *Flavobacterium* sp. strain GJW24 improved drought and drought-related salt stress resistance in *Arabidopsis* and *Brassica* plants. The abiotic stress resistance phenotype conferred by GJW24 increases the possibility that GJW24 can be utilized as a biological material to positively regulate plant growth and yield under such unfavorable environments.

## Materials and methods

### Plant materials and culture conditions

*Arabidopsis* (*A. thaliana*) ecotype Col-0 and cabbage (*Brassica campestris* L. ssp. pekinensis, cultivar; ‘Chun Yeon Gold’) were used as the wild types in this study. The surfaces of the plant seeds were sterilized, and the seeds were grown on 1× MS plate supplemented with 0.8% bacto agar and 1% sucrose (pH 5.8). The seeds were kept for 7 d in a growth chamber at 23°C under a 16 h light/8 h dark cycle. Then, the resulting seedlings were moved to into a pot filled with horticultural soil mix (cocopeat 51.5%, peat moss 10%, vermiculite 13%, perlite 15%, zeolite10%, humic acid 0.1%, fertilizer 0.4%) and kept in a growth chamber at 23°C under a 16 h light/8 h dark cycle for further analysis.

### Bacterial growth conditions and plant inoculation

The *Flavobacterium* sp. strain GJW24 was obtained from Gotjawal soil, Jeju Island, South Korea (Kim et al., 2018). GJW24 was incubated on R2A plates overnight at 28°C. The resulting microbes were adjusted to an OD_600_ of 0.02~0.03 using MS solution. Next, one milliliter of the bacterial suspension was added to the region around the growth areas of *Arabidopsis* and *Brassica*.

### Assay for drought/salt stress resistance in *Arabidopsis*

Assays for drought and salt stress resistance in *Arabidopsis* were performed using the MS liquid (mock) or GJW24-treated plants. For the drought tolerance assay, 8-day-old plants were exposed to mock or GJW24. Seven days after treatment, they were exposed to water deficiency (drought stress) for 14 d, followed by re-watering for 4 d. The assays were conducted with four biological replicates. For each assay, at least ten plants were used for mock or GJW24 treatment. The assay for salt stress resistance was conducted by supplying 50 mL of water or 150 mM NaCl solution to the soil 7 d after mock or GJW24 treatment, three times for 6 d at 3-d intervals. Survival rates were monitored 3 d after the last day of NaCl treatment. The assays were conducted with six biological replicates. In four combinations of GJW24 and salt stress treatments for each assay, at least eight plants were used for each combination.

### Assay for drought/salt stress resistance in *Brassica*

For the drought tolerance assay for *Brassica*, 8-day-old *Brassica* plants were exposed to MS liquid (mock) or GJW24. Seven days after treatment, the plants were exposed to water deficiency (drought stress) for 10 d, followed by re-watering for 6 d. The assays were conducted with four biological replicates. For each assay, at least six plants were used for mock or GJW24 treatment. The assay for salt stress resistance was performed by supplying 50 mL of water or 150 mM NaCl solution to the soil 7 d after mock or GJW24 treatment, eight times at 3-d intervals. Survival rates were monitored 1 d after the last day of NaCl treatment. For each assay, at least eleven plants were used for mock or GJW24 treatment.

### Measurement of stomatal aperture

Eight-day-old *Arabidopsis* plants were exposed to mock or GJW24. Seven days after treatment, detached leaves from mock- or GJW24-treated plants were floated in stomatal opening solution containing 10 mM MES-KOH (pH 6.15), 50 mM KCl and 10 mM CaCl_2_ for 2.5 h in the light (Lee et al., 2013). Then, the samples were additionally incubated in the same buffer with 0 or 20 μM ABA for 2.5 h in the light. The stomatal apertures from the abaxial part of the treated leaves were measured using a microscope (DM750, Leica) and the ImageJ program, as described by Kim et al. (2020).

### Measurement of chlorophyll concentration

The total chlorophyll contents of the mock- or GJW24-treated samples under salt stress were quantified as described by Woo et al. (2020). The extracts were prepared using 80% acetone, and were then maintained in the dark for 30 min. The assays were conducted with six biological replicates. The chlorophyll content was quantified using the supernatants from centrifugation, and determined as described by Woo et al. (2020).

### RNA isolation and RT-qPCR analysis

Total RNA was extracted using TRIzol reagent (Ambion). From one microgram of total RNA, complementary DNA (cDNA) was synthesized using RevertAid™ reverse transcriptase (Fermentas). Quantitative real-time PCR (qRT-PCR) was performed as described by Woo et al. (2020). The qRT-PCR was performed using the Solg™ 2 × real-time PCR Smart Mix (SolGent) and analyzed with the Rotor-Gene Q system (Qiagen). The transcript amounts of each gene were monitored using the comparative cycle threshold (CT) method, which was normalized against the transcripts from the reference genes; *Arabidopsis ACTIN2* or *Brassica ACT7* (Schmittgen and Livak, 2008). The DNA sequences for RT-qPCR analysis are listed in **Table S1**. The qRT-PCR analyses were performed with at least six biological replicates.

### Transcriptome sequencing analysis

For transcriptome sequencing analysis, eight-day-old *Arabidopsis* plants were exposed to mock or GJW24. Total RNAs were independently extracted from the entire *Arabidopsis* plants (8 plants for each treatment) with three biological replicates 7 d after mock or GJW24 treatment. The replicates were not pooled per treatment. The analysis was done by MacroGen Inc. (Korea). To evaluate total RNA integrity, samples were run on a TapeStation RNA Screentape (Agilent Technologies). The RNA library was generated using RNA with RNA integrity number higher than 7.0. A total RNA library (0.5 μg) for each sample was produced using the Illumina TruSeq Stranded Total RNA Library Prep Plant Kit (Illumina, Inc.). After removing the total ribosomal RNA, the messenger RNA was fragmented using divalent cations at elevated temperatures. The resulting fragmented mRNAs were converted into first-strand cDNA with random primers and SuperScript II reverse transcriptase (Invitrogen). After second-strand cDNA synthesis using DNA Polymerase I, RNase H and dUTP, the resulting cDNA fragments go through an end repair process, the addition of a single ‘A’ base, and then ligation of the adapters. After purification, the products were enriched using PCR to construct the final cDNA library. Libraries were quantified using KAPA Library Quantification kits for Illumina Sequencing platforms (Kapa Biosystems) and qualified using a TapeStation D1000 ScreenTape (Agilent Technologies). Paired-end (2×100 bp) sequencing of indexed libraries was conducted using an Illumina NovaSeq system (Illumina, Inc.), by MacroGen Inc. (Korea). After removing adapter sequences and trimming bases with poor base quality using Trimmomatic v0.38, the cleaned reads were aligned to TAIR 10.1 with HISAT v2.1.0 (Kim et al., 2015), based on the HISAT and Bowtie2 implementations. The reference genome sequence and the gene annotation data were retrieved from NCBI Genome assembly and NCBI RefSeq database, respectively. Aligned data were sorted and indexed with SAMtools v 1.9, and the transcripts were assembled and quantified with StringTie v2.1.3b (Pertea et al., 2015; Pertea et al., 2016). Gene-level and transcript-level quantification were calculated with raw read count, FPKM and TPM.

### Differential gene expression analysis

Among total 38,338 genes, 21,834 genes with non-zero counts in all replicates at least one period group were retrieved during the QC step. The fold change and the statistical significance of DEGs was investigated by exactTest using edgeR (Robinson et al., 2010). Among the 21,834 genes analyzed, the expression of the transcript that satisfied with FC ≥ 2 and p < 0.05 at all comparisons was considered as significantly up-regulated genes. All p-values are adjusted using Benjamini-Hochberg algorithm to control false discovery rate. To investigate characteristic biological attributes of the genes upregulated by GJW24 treatment, Gene Ontology (GO) analysis was performed with the functional annotation tool on DAVID (https://david.ncifcrf.gov/tools.jsp), and the GJW24-upregulated genes were assigned to different categories for biological processes (BPs) and molecular functions (MFs). The inducibility of GJW24-upregulated genes under drought stress were checked using the database from the Arabidopsis eFP Browser (Winter et al., 2007).

### Phylogenetic analysis

To generate a phylogenetic tree based on the 16s rRNA sequences, the 16s rRNA sequences of 17 *Flavobacterium* type strains, which exhibited the highest similarity to that of GJW24 based on a BLAST search, and 9 type strains from other genera in the family *Flavobacteriaceae* were obtained from ‘The List of Prokaryotic names with Standing in Nomenclature’ (LPSN) (https://lpsn.dsmz.de/text/approved-lists). The 16s rRNA sequences were aligned with ClustalW running in the MEGA11 program (Tamura et al., 2021). A rooted phylogenetic tree was generated with the MEGA11 using the maximum-likelihood method. The bootstrap method was performed to assess statistical significance of the tree using 1000 replicates.

## Results and discussion

### Identification of a *Flavobacterium* involved in drought tolerance process in *Arabidopsis*

**Figure 1:**
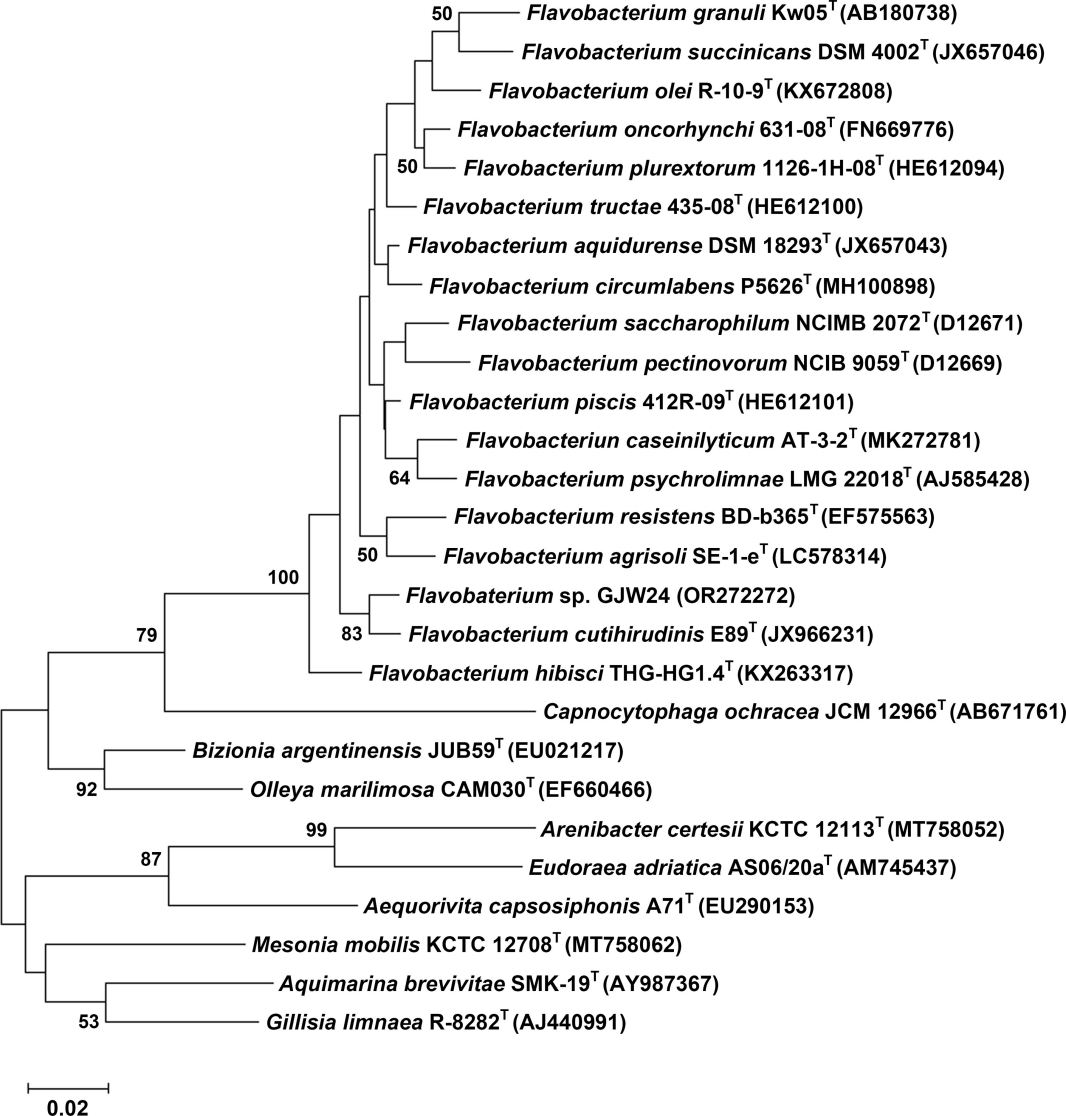
A phylogenetic tree among *Flavobacterium* sp. strain GJW24, diverse *Flavobacterium* type strains, and type strains from other genera in the family *Flavobacteriaceae*, based on their 16S rRNA gene sequences. The 16s rRNA sequences were obtained from LPSN and aligned with ClustalW running in the MEGA11 program. A rooted phylogenetic tree was generated with the MEGA11 using the maximum-likelihood method. The bootstrap method was performed to assess statistical significance of the tree using 1000 replicates. Bootstrap values (>50%) are indicated at the nodes.

Approximately 500 soil microbes, which included a unique microbial community, have previously been obtained from Gotjawal, Jeju Island (Kim et al., 2018; Woo et al., 2020). Through a preliminary screening process to select PGPR involved in drought stress resistance in *Arabidopsis*, several microbes whose applications resulted in the upregulation of drought resistance marker genes in *Arabidopsis* were identified (Woo et al., 2020). The 16S rRNA gene of the selected candidate exhibited the highest similarity to those of diverse *Flavobacterium* strains based on a BLAST search, showing that it belongs to the genus *Flavobacterium*. Therefore, the strain was named *Flavobacterium* sp. strain GJW24. The 16S rRNA gene sequence of GJW24 has been uploaded in GenBank under accession number OR272272. To examine how closely related GJW24 is to other *Flavobacterium* strains that have already been characterized, the 16s rRNA sequences of type strains from diverse *Flavobacteria* and other genera in the family *Flavobacteriaceae* were obtained from LPSN, and a phylogenetic tree based on the 16s rRNA sequences of GJW24 and the selected type strains was generated (**Figure 1**). Although the *Flavobacterium* genus has been reported as a PGPR group, studies on its biological contribution to the drought stress response in plants are highly limited (Gontia-Mishra et al., 2016; Kim et al., 2020); therefore, the biological function of GJW24 was further analyzed in this study.

### Improved drought/salt stress resistance in *Arabidopsis* and *Brassica* following GJW24 application

**Figure 2:**
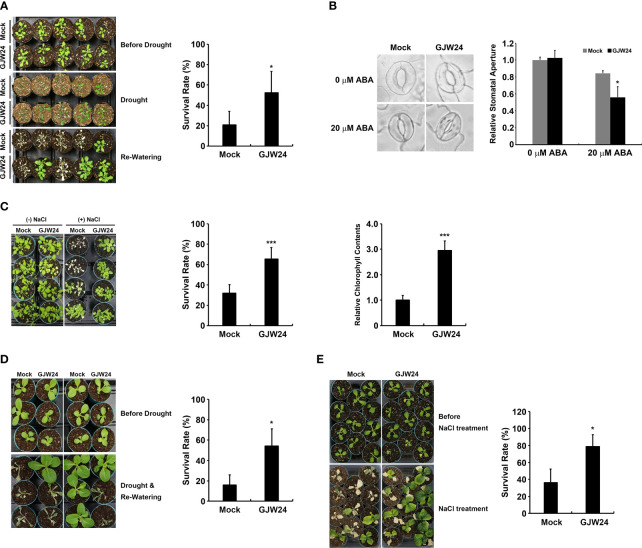
Phenotypic analysis of *Arabidopsis* and *Brassica* plants in response to drought and salt stressors in the presence and absence of GJW24. **(A)** Increased drought tolerance of *Arabidopsis*. Eight-day-old *Arabidopsis* plants were treated with mock or GJW24 and watered regularly for 1 week. The resulting plants were exposed to dehydration stress for 14 d, followed by re-watering for 4 (d) Error bars indicate the standard deviation (SD) of four biologically independent experiments. A Student’s t-test was conducted to identify statistically significant differences; *P < 0.05. **(B)** Stomatal movements of *Arabidopsis* with or without GJW24, in the absence or presence of ABA. Eight-day-old *Arabidopsis* plants were treated with mock or GJW24. Seven days after treatment, detached leaves from mock- or GJW24-treated plants were prepared to measure the stomatal apertures in the absence or presence of ABA. Error bars indicate the SD of three biologically independent experiments (at least 40 stomata for each biological sample); *P < 0.05. **(C)** Enhanced salt resistance in *Arabidopsis* following GJW24 application. Eight-day-old plants were treated with mock or GJW24. Salt stress resistance was observed by introducing 50 mL of water or 150 mM NaCl solution to the soil 7 d after GJW24 treatment, three times for 6 d at 3-d intervals. The survival rates (n = 6) and the chlorophyll contents (n = 6) under salt stress were determined 3 and 4 d after the final salt treatment, respectively; ***P < 0.001. **(D)** Improved drought resistance in *Brassica* following GJW24 application. Eight-day-old *Brassica* plants were treated with mock or GJW24 and watered regularly for 1 week. The resulting plants were subjected to dehydration stress for 10 d, followed by re-watering for 6 (d) Error bars indicate the SD of four biologically independent experiments; *P < 0.05. **(E)** Enhanced salt tolerance in *Brassica* following GJW24 application. Ten-day-old *Brassica* plants were treated with mock or GJW24. Salt stress tolerance was observed by introducing 50 mL of water or 150 mM NaCl solution to the soil 7 d after mock or GJW24 treatment, eight times at 3-d intervals. The survival rates under salt stress were determined 1 d after the final salt treatment (n = 6); ***P < 0.001.

To examine the potential functional role of GJW24 in the drought stress response, a phenotypic analysis of drought resistance in *Arabidopsis* was performed in the presence and absence of GJW24. The survival rate of GJW24-treated plants under drought condition was more than 2.5-fold higher than that of the mock-treated plants (**Figure 2A**). Given that tolerance against drought stress is largely achieved through the modulation of the stomatal aperture in plants, the effect of GJW24 on stomatal aperture regulation was further monitored. Although the difference of the stomatal apertures between mock-treated and GJW24-treated plants was not detected in the absence of ABA, the stomatal closure of GJW24-treated plants was significantly enhanced compared to mock-treated plants in the presence of ABA, indicating that GJW24 is involved in the promotion of ABA-mediated stomatal closure (**Figure 2B**). ABA is a crucial phytohormone that mediates drought tolerance by promoting stomatal closure and modulating drought-responsive genes (Takahashi et al., 2020). Thus, the enhancement of ABA-dependent stomatal closure in *Arabidopsis* by GJW24 (**Figure 2B**) shows that the regulation of the ABA signal transduction pathway is involved in the GJW24-mediated drought tolerance process. Collectively, these findings indicate that GJW24 improves tolerance against drought stress and that this process may be accomplished by minimizing water loss from transpiration, which results, at least in part, from the promotion of ABA-mediated stomatal closure. As salt stress increases endogenous ABA levels in plants, the plant salt stress response has been reported to largely overlap with the ABA-mediated drought stress response (Shinozaki and Yamaguchi-Shinozaki, 2007). Therefore, to confirm the GJW24-induced improvement in drought tolerance, the effect of GJW24 on salt stress resistance was examined. Similar to drought stress, the survival rate and chlorophyll content of GJW24-treated plants were approximately two- and three-fold higher than those of mock-treated ones under salt stress, respectively (**Figure 2C**), indicating that GJW24 application enhanced salt stress tolerance. To determine whether the GJW24-induced drought/salt stress resistance in *Arabidopsis* could be applied to other plants, the effect of GJW24 on abiotic stress resistance in *Brassica*, a crop most closely related to *Arabidopsis*, was investigated (Lagercrantz et al., 1996; Woo et al., 2020). Similar to its effect on *Arabidopsis*, GJW24 improved drought/salt stress tolerance in *Brassica*. Under drought and salt stress, the survival rate of GJW24-treated *Brassica* plants was more than three- and two-fold higher than that of mock-treated plants, respectively (**Figures 2D, E**). GJW24-induced improved drought/salt stress tolerance in *Arabidopsis* and *Brassica* increases the possibility that GJW24 may be utilized as a biological resource to facilitate plant growth under drought/salt stress condition.

### GJW24 increases upregulation of a large portion of drought/salt/osmotic stress−inducible genes

**Figure 3:**
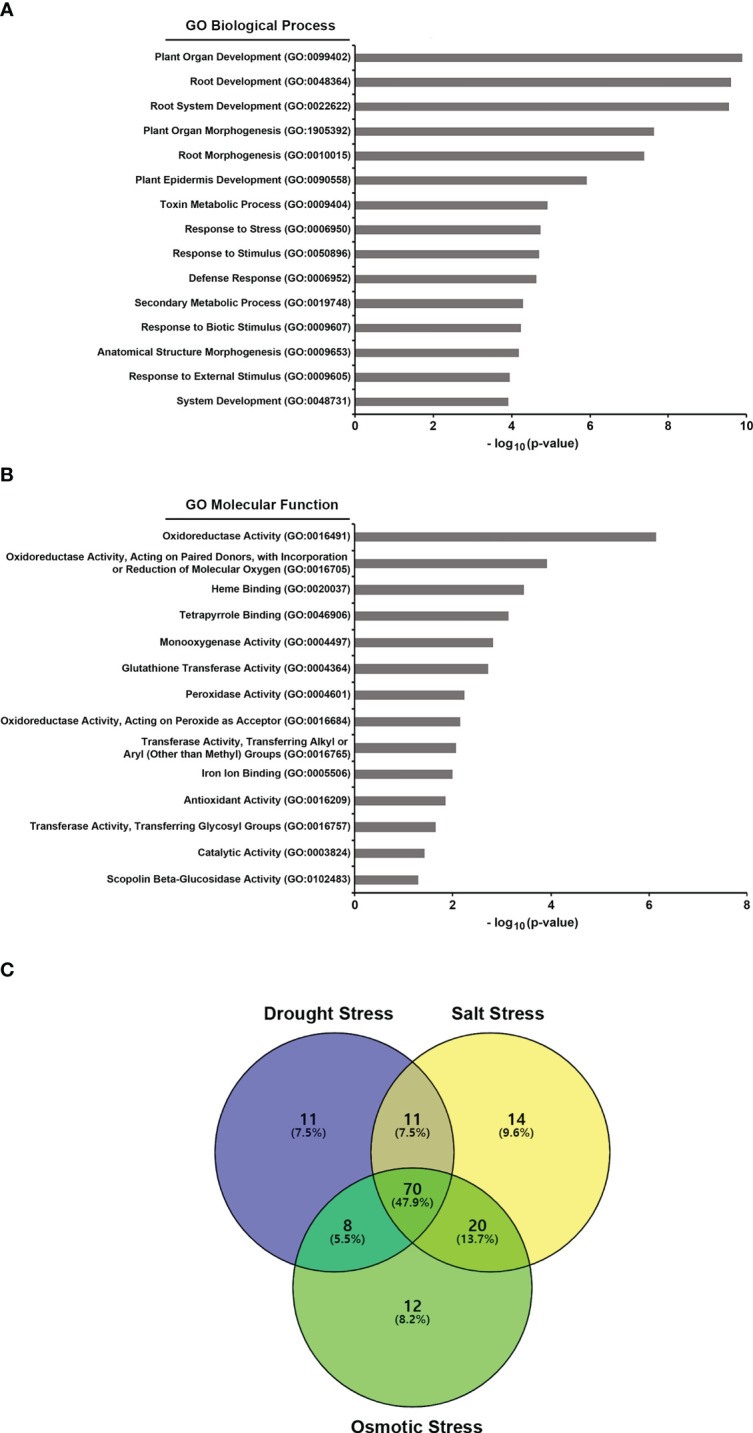
Gene ontology (GO) analysis of the GJW24-upregulated genes and Venn diagram of the genes induced by drought and drought-related stressors among the GJW24-upregulated genes. **(A, B)** GO analysis of biological processes **(A)** and molecular functions **(B)** for GJW24-upregulated genes. GO terms were identified by the functional annotation tool on DAVID. **(C)** Three-set Venn diagram of the overlap of drought stress-, salt stress- and osmotic stress-inducible genes in the GJW24-upregulated gene expression datasets. Among 251 genes that showed increased gene expression by at least twofold in the GJW24-treated samples, drought stress- (100 genes), salt stress- (115 genes), and osmotic stress-inducible genes (110 genes), which exhibited more than two-fold increases by each stressor (based on Arabidopsis eFP Browser) were retrieved and used generate Venn diagram. Courtesy: Oliveros, J.C. (2007-2015) Venny. An interactive tool for comparing lists with Venn diagrams: http://bioinfogp.cnb.csic.es/tools/venny/index.html.

To understand the action mechanism of GJW24 in drought/salt stress resistance in *Arabidopsis*, transcriptome sequencing analysis of *Arabidopsis* was performed in the presence and absence of GJW24. Among the 21,834 genes analyzed, the expression of 251 showed at least a two-fold increase in the GJW24-treated samples compared to the mock-treated samples (**Tables 1**, **S2**). To investigate characteristic biological attributes of the genes upregulated by GJW24 treatment, Gene Ontology (GO) analysis was done with the functional annotation tool on DAVID (https://david.ncifcrf.gov/tools.jsp), and the GJW24-upregulated genes were assigned to different categories for biological processes (BPs) (**Figure 3A** and **Table S3**) and molecular functions (MFs) (**Figure 3B** and **Table S4**). For BP, the genes were highly related to categories involved in root system architecture and development. Furthermore, GO terms related to ‘response to stress’ were notably enriched. To investigate whether the GJW24-conferred drought resistance phenotype in *Arabidopsis* is functionally associated with the altered expression patterns observed in GJW24-treated plants, the inducibility of GJW24-upregulated genes under drought stress was monitored using the database from the Arabidopsis eFP Browser (Winter et al., 2007). Based on information from the Arabidopsis eFP Browser, for each gene, the expression values from drought-treated samples (approximately 10% fresh weight loss) at various time points (0.25, 0.5, 1, 3, 6, 12 or 24 h) were divided by the values from untreated samples at the same time points. The maximum fold increase for each gene was then retrieved. Of the total 251 GJW24-specific genes, 100 (39.8%) exhibited increased expression patterns by more than twofold by drought stress, indicating that the application of GJW24 upregulated several drought-inducible genes (**Tables 1**, **S2**). Given that drought stress responses in plants largely overlaps with the responses to salt and osmotic stressors (Zhu, 2002), the inducibility of the GJW24-upregulated genes to salt and osmotic stress was also investigated based on information from the Arabidopsis eFP Browser. For each gene, the expression values from 150 mM NaCl-treated samples (salt) at various time points (0.5, 1, 3, 6, 12 or 24 h) or 300 mM mannitol-treated samples (osmotic) at various time points (0.5, 1, 3, 6, 12 or 24 h) were divided by the values from untreated samples at the same time points. The maximum fold increase for each gene was then obtained. Similar to drought-inducible genes, 115 (45.8%) and 110 (43.8%) of the total 251 GJW24-specific genes exhibited upregulated expression of more than twofold by salt and osmotic stress, respectively (**Tables 1**, **S2**). Moreover, 47.9% (70 genes) of the 146 genes induced by drought, salt, or osmotic stress among the GJW24-upregulated genes were commonly upregulated by these three stressors (**Figure 3C**). Collectively, these findings indicate that GJW24 upregulated a large portion of drought- and drought-related stress-inducible genes in *Arabidopsis*. Previous researches have shown that drought resistance traits are functionally associated with the upregulation of drought-inducible genes in plants (Kim et al., 2020; Woo et al., 2020); therefore, GJW24-induced enhanced drought/salt stress tolerance may result from the overall enhancement of the upregulation of genes induced by drought and drought-related stressors. The GO analysis results revealed that the GJW24-upregulated genes were highly assigned to the categories related to root development. Several PGPR have been demonstrated to modify root system architecture and modulate root development (Vacheron et al., 2013; Zamioudis et al., 2013; Zhou et al., 2016), which supports our suggestion that GJW24 can play a role as a PGPR. Furthermore, many reports indicate that several PGPR involved in root development and growth contribute to enhanced plant drought stress tolerance (Cohen et al., 2015; Vurukonda et al., 2016; Jochum et al., 2019; Woo et al., 2020; Zia et al., 2021), showing that the GJW24-induced improvement of drought resistance may be associated with its possible role in root system architecture and development for efficient water uptake.

**Table 1:** Changes in expression patterns of abiotic stress-inducible genes following GJW24 application.

		Upregulated genes by GJW24 treatment^a^
Total number of genes		251
The number of genes induced by drought stress	More than two times^b^	100 (39.8%)
	More than five times^b^	36 (14.3%)
	More than ten times^b^	18 (7.1%)
The number of genes induced by salt stress	More than two times^b^	115 (45.8)
	More than five times^b^	53 (21.1%)
	More than ten times^b^	23 (9.1%)
The number of genes induced by osmotic stress	More than two times^b^	110 (43.8%)
	More than five times^b^	52 (20.7%)
	More than ten times^b^	30 (12.0%)

a Genes with increased expression rates by more than twofold in GJW24-treated samples compared to mock-treated samples.

b Increased gene expression rates following abiotic stress based on information from Arabidopsis eFP Browser. Detailed information is provided in **Table S2**.

### GJW24-mediated expression pattern of several drought- and drought-related stress-responsive genes in *Arabidopsis* and *Brassica*

**Figure 4:**
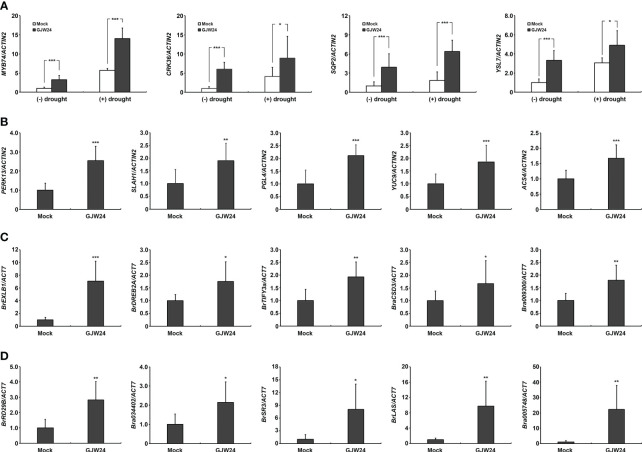
Induction of several drought- and salt-inducible genes in response to GJW24 treatment in *Arabidopsis* and *Brassica*. **(A)** Validation of the GJW24- and drought-upregulated genes from transcriptome sequencing analysis and Arabidopsis eFP Browser. Eight-day-old *Arabidopsis* seedlings were exposed to mock or GJW24. After 7 d, they were taken out of the pots in which they were growing. After checking the fresh weight, they were placed on filter paper until approximately 20% of their total fresh weight was lost or maintained without dehydration. The average expression value of each gene from mock-treated samples without drought stress was set to 1.0. Values indicate means ± SD (n ≥ 6). A Student’s t-test was conducted to identify statistically significant differences; *P < 0.05; ***P < 0.001. **(B)** Upregulation of various salt-inducible genes in *Arabidopsis* by GJW24 treatment under salt stress. Eight-day-old *Arabidopsis* seedlings were exposed to mock or GJW24. The salt stress resistance assay was conducted by adding 50 mL of 200 mM NaCl solution or water to the soil 7 d after mock or GJW24 treatment, twice at 3-d intervals for 3 (d) The samples were obtained 3 d after the last day of NaCl treatment. The average expression value of each gene from mock-treated samples was set to 1.0. Values indicate means ± SD (n ≥ 6); **P < 0.01; ***P < 0.001. **(C)** Upregulation of various drought-inducible genes in *Brassica* by GJW24 treatment under drought stress. Eight-day-old *Brassica* seedlings were treated with mock or GJW24. After 7 d, they were taken out of the pots in which they were growing. After checking the fresh weight, they were placed on filter paper until approximately 20% of their total fresh weight was lost. Values indicate means ± SD (n ≥ 6); *P < 0.05; **P < 0.01; ***P < 0.001. **(D)** Upregulation of various salt-inducible genes in *Brassica* by GJW24 treatment under salt stress. Eight-day-old *Brassica* seedlings were treated with mock or GJW24. The salt stress resistance assay was conducted by adding 50 mL of water or 300 mM NaCl solution to the soil 7 d after mock or GJW24 treatment, twice at a 3-day interval for 3 (d) The samples were obtained 3 d after the last day of NaCl treatment. Values indicate means ± SD (n ≥ 6); *P < 0.05; **P < 0.01.

The GJW24 treatment led to an overall enhancement of the upregulation of drought stress-inducible genes (**Table 1**). To confirm the list of GJW24-upregulated genes based on the information from the transcriptome sequencing analysis and their drought responsiveness using the Arabidopsis eFP Browser, several genes whose expression was increased by more than two folds by both GJW24 and drought stress were retrieved from **Table S2**, and their expression patterns were validated using qPCR (**Figure 4A**). The four selected genes, *MYB74*, *CRK36*, *SQP2*, and *YSL7*, showed similar GJW24- and drought-inducible patterns to those shown in the transcriptome sequencing analysis and the Arabidopsis eFP Browser database, supporting the reliability of the gene expression profiling results related to both GJW24- and drought-inducible genes (**Figure 4A**). Furthermore, GJW24 enhanced the upregulation of the selected genes under drought stress (**Figure 4A**). The expression levels of *PERK13*, *SLAH1*, *PGL4*, *YUC9*, and *ACS4*, which were selected from the list of GJW24- and salt-up-regulated genes, were also increased by GJW24 under salt stress (**Figure 4B**). Next, to determine whether the GJW24-induced drought- and salt-stress resistant phenotypes in *Brassica* were functionally connected to the expression of the corresponding stress-responsive genes, the expression patterns of drought- or salt-inducible genes in *Brassica* were examined with or without GJW24 treatment under stress conditions. GJW24 increased the expression of selected drought− (*BrEXLB1*, *BrDREB2A*, *BrTIFY3a*, *BraCSD3* and *Bra009300*) and salt−inducible genes (*BrRD29B*, *Bra034402*, *BrSR3*, *BrLAS* and *Bra005748*) in *Brassica* plants, similar to *Arabidopsis* (**Figures 4C, D**) (Yadav et al., 2015; Saha et al., 2016; Li et al., 2018; Yoon et al., 2018; Alam et al., 2019; Jin et al., 2019; Verma et al., 2019; Muthusamy et al., 2020). Collectively, these gene expression results indicate that GJW24 was involved in the upregulation of various drought- and salt-inducible genes in *Brassica* and *Arabidopsis*, suggesting that the modulation of drought and salt stress resistance by GJW24 may be achieved, at least in part, by regulating the expression of genes associated with drought and salt resistance in both plant species.

## Conclusion

Compared to chemical fertilizers or pesticides, which cause environmental pollution, the use of environmentally friendly PGPR may contribute to improved plant growth in a more desirable manner. Here, GJW24 was selected from Gotjawal soil as a useful PGPR that increased the drought resistance of *Arabidopsis* and *Brassica*. Although the potential value of GJW24 as a biological agent in agriculture has been demonstrated in this study, the detailed mechanism by which GJW24 enhances plant drought tolerance still requires further investigation. Given that various secondary metabolites and phytohormones (IAA, cytokinin, ABA, and GA) produced by PGPR have been reported to aid the adaptation of plants to abiotic stress conditions (Ma et al., 2020), predicting the list of secondary metabolites and phytohormones that GJW24 can produce from the whole genome sequencing of GJW24 may be useful for a better understanding of the function of GJW24. The finding that several auxin- and ABA-responsive genes are included in the list of GJW24-upregulated *Arabidopsis* genes increases the possibility that GJW24 may utilize the regulation of plant hormone signaling as a strategy to confer drought tolerance in plants (**Table S2**).

## Data availability statement

The data presented in this study are deposited in the NCBI repository (https://www.ncbi.nlm.nih.gov/), accession number OR272272.

## Author contributions

HK: Conceptualization, Data curation, Funding acquisition, Investigation, Validation, Writing – review & editing, Formal Analysis. O-GW: Data curation, Formal Analysis, Investigation, Validation, Writing – review & editing, Writing – original draft. JBK: Data curation, Formal Analysis, Investigation, Validation, Writing – review & editing. S-YY: Data curation, Validation, Writing – review & editing. J-SK: Writing – review & editing, Resources. WJS: Writing – review & editing, Data curation, Formal Analysis. J-YH: Data curation, Formal Analysis, Writing – review & editing. J-HL: Data curation, Writing – review & editing, Conceptualization, Funding acquisition, Investigation, Project administration, Supervision, Validation, Writing – original draft.
