# Supplementary material for: Flavobacterium sp. strain GJW24 ameliorates drought resistance in Arabidopsis and Brassica
Source: Front Plant Sci. 2023 Oct 13;14:1257137. doi: 10.3389/fpls.2023.1257137 (PMC10613084; doi:10.3389/fpls.2023.1257137)
Supplement: Supplementary file 1 [file Table_1.docx]

**Table S1** The information of the primer pairs used for RT-qPCR analysis.

| *Arabidopsis* Gene Name | Gene Identification Number^a^ | DNA Sequence (5’ – 3’) |
| --- | --- | --- |
| *ACTIN2*_Forward Primer | At3g18780 | CAAGGCCGAGTATGATGAGG |
| *ACTIN2*_Reverse Primer | At3g18780 | GAAACGCAGACGTAAGTAAAAAC |
| *MYB74*_ Forward Primer | At4g05100 | CCAATGAAGACGACGGTCCA |
| *MYB74*_ Reverse Primer | At4g05100 | CACCATGGAGAGGGAGAACC |
| *CRK36*_ Forward Primer | At4g04490 | TGGGATGGCGAGGTTGTTTA |
| *CRK36*_ Reverse Primer | At4g04490 | TTGTCCATGTCTCACGTACTCC |
| *SQP2*_ Forward Primer | At5g24140 | GAATGCGACAAGGCTGCTTC |
| *SQP2*_ Reverse Primer | At5g24140 | AAGTGGCCCATGGACGTAAG |
| *YSL7*_ Forward Primer | At1g65730 | CGGTTTATCCCGCTCCCAAT |
| *YSL7*_ Reverse Primer | At1g65730 | GAAGAGTACGCATCGGCCTT |
| *PERK13*_ Forward Primer | At1g70460 | AGTGAAGGGGACATGGGAGA |
| *PERK13*_ Reverse Primer | At1g70460 | CCTTTCCGGGAGTCTTGGAC |
| *SLAH1*_ Forward Primer | At1g62280 | GGGCTTACTCGTTCCCACTC |
| *SLAH1*_ Reverse Primer | At1g62280 | GCATTAAGCCAGAACCGACC |
| *PGL4*_ Forward Primer | At5g24410 | AACCGCCTCCGAAGAGAATC |
| *PGL4*_ Reverse Primer | At5g24410 | TACGTCAGCTGTGAGCCAAG |
| *YUC9*_ Forward Primer | At1g04180 | AAGACGCCGGTTCTTGACAT |
| *YUC9*_ Reverse Primer | At1g04180 | GACCATCTACGAGCTCCACG |
| *ACS4*_ Forward Primer | At2g22810 | GTTGGGTCTAGAGGCCATCG |
| *ACS4*_ Reverse Primer | At2g22810 | TCTTCACAATGGCACGACGA |
| *Brassica* Gene Name | Gene Identification Number^a^ | DNA Sequence (5’ – 3’) |
| *ACT7*_Forward Primer | LOC103850356 | CTACGAGTTACCTGATGGAC |
| *ACT7*_Reverse Primer | LOC103850356 | ATGATGGAGTTGTAAGTTGTCTC |
| *BrEXLB1*_Forward Primer | LOC106437435 | TATGGAGAGGGACATGGCAC |
| *BrEXLB1*_Reverse Primer | LOC106437435 | CCTCTGGTACTCAACGTCGA |
| *BrDREB2A*_Forward Primer | LOC103855625 | AGCCTGCATCGCTATCTGTT |
| *BrDREB2A*_Reverse Primer | LOC103855625 | CGCTAGACATTGGCTCTGG |
| *BrTIFY3a*_Forward Primer | LOC103845633 | CTCTCTCAAGCACATCCAAT |
| *BrTIFY3a*_Reverse Primer | LOC103845633 | TTCTTGTCTGAAGCAGGGTA |
| *BraCSD3*_Forward Primer | LOC103845867 | CTGCAACTCTACTGGACCTCACT |
| *BraCSD3*_Reverse Primer | LOC103845867 | GTCTGATCCAGCGAAAATGTTAC |
| *Bra009300*_Forward Primer | LOC103847100 | AGAGCTTGAGGACACCAGAGA |
| *Bra009300*_Reverse Primer | LOC103847100 | GAAGCTGCACAGATCCGAGA |
| *BrRD29B*_Forward Primer | LOC103857129 | ATGGTGGGGAAGGTTAAAGG |
| *BrRD29B*_Reverse Primer | LOC103857129 | GTGGAGCCAAGTGACTGTGA |
| *Bra034402*_Forward Primer | LOC103868807 | CGAGGTGAACATGACATTGGC |
| *Bra034402*_Reverse Primer | LOC103868807 | CGTGCTCATCTTGGTAGCGA |
| *BrSR3*_Forward Primer | LOC103871643 | ATGAGTAGCAGCCGATGGAA |
| *BrSR3*_Reverse Primer | LOC103871643 | TGCATCACGAGGATCTTCAA |
| *BrLAS*_Forward Primer | LOC103871035 | CATAACCGGATGTGGTCG |
| *BrLAS*_Reverse Primer | LOC103871035 | AAGTGGACGCAGTTGACG |
| *Bra005748*_Forward Primer | LOC103855501 | CTACCTCCTGGGGGCTTG |
| *Bra005748*_Reverse Primer | LOC103855501 | CACACTCGGCAAGTCAGTTTCTGAA |

^a^ Gene Identification Number was based on information from NCBI.
