# Supplementary material for: Flavobacterium sp. strain GJW24 ameliorates drought resistance in Arabidopsis and Brassica
Source: Front Plant Sci. 2023 Oct 13;14:1257137. doi: 10.3389/fpls.2023.1257137 (PMC10613084; doi:10.3389/fpls.2023.1257137)
Supplement: Supplementary file 2 [file Table_2.docx]

**Table S2** List of upregulated genes by GJW24, based on the transcriptome sequencing.

| AGI | Gene Name | Fold Induction by GJW24^a^ | Gene Description^b^ | Fold Induction by abiotic stress, based on information from Arabidopsis eFP Browser^c^ | | |
| --- | --- | --- | --- | --- | --- | --- |
|  |  |  |  | Drought | Salt | Osmotic |
| AT1G01580 | *FRO2* | 3.58 | ferric reduction oxidase 2 | N/A^d^ | N/A | N/A |
| AT1G01680 | *PUB54* | 3.55 | plant U-box 54 | N/A | N/A | N/A |
| AT1G01750 | *ADF11* | 2.80 | actin depolymerizing factor 11 | 3.71 | 5.1 | 6.56 |
| AT1G02920 | *GSTF7* | 2.33 | glutathione S-transferase 7 | 10.15 | 4.94 | 30.45 |
| AT1G02930 | *GSTF6* | 2.09 | glutathione S-transferase 6 | 10.15 | 4.94 | 30.45 |
| AT1G03580 |  | 2.28 |  | N/A | N/A | N/A |
| AT1G04180 | *YUC9* | 3.12 | YUCCA 9 | 2.68 | 2.02 | 4.33 |
| AT1G04197 |  | 2.26 | other RNA (snoRNA) | N/A | N/A | N/A |
| AT1G05400 |  | 3.46 | hypothetical protein | 2.17 | 3.49 |  |
| AT1G05660 |  | 3.85 | pectin lyase-like superfamily protein | 2.31 | 2.75 | 2.67 |
| AT1G05680 | *UGT74E2* | 5.80 | uridine diphosphate glycosyltransferase 74E2 | 6.51 | 150.22 | 27.46 |
| AT1G06090 |  | 2.78 | fatty acid desaturase family protein | 2.09 |  |  |
| AT1G06120 |  | 3.00 | fatty acid desaturase family protein | 2.09 |  |  |
| AT1G06473 |  | 3.37 | other RNA (lncRNA) | N/A | N/A | N/A |
| AT1G08990 | *PGSIP5* | 2.31 | plant glycogenin-like starch initiation protein 5 | 2.85 | 3.91 | 8.52 |
| AT1G13550 |  | 5.55 | hypothetical protein (DUF1262) | N/A | N/A | N/A |
| AT1G14540 | *PER4* | 3.57 | peroxidase superfamily protein | 7.25 | 122.55 | 5.04 |
| AT1G14880 | *PCR1* | 4.71 | PLANT CADMIUM RESISTANCE 1 | 8.2 | 4.43 | 30.69 |
| AT1G14960 |  | 4.55 | polyketide cyclase/dehydrase and lipid transport superfamily protein |  |  |  |
| AT1G19371 | *MIR169h* | 2.41 | microRNA ath-MIR169h precursor | N/A | N/A | N/A |
| AT1G19396 |  | 4.41 | hypothetical protein | N/A | N/A | N/A |
| AT1G19620 |  | 3.56 | transmembrane protein |  | 2.71 | 2.85 |
| AT1G21110 | *IGMT3* | 2.85 | O-methyltransferase family protein | 13.91 | 5.31 | 54.71 |
| AT1G23040 |  | 2.37 | hydroxyproline-rich glycoprotein family protein |  | 3.97 | 7.14 |
| AT1G25460 |  | 2.80 | NAD(P)-binding Rossmann-fold superfamily protein |  |  |  |
| AT1G26380 |  | 4.48 | FAD-binding Berberine family protein | 10.36 | 14.48 | 438.09 |
| AT1G27020 |  | 2.84 | plant/protein | N/A | N/A | N/A |
| AT1G28815 |  | 3.06 | hypothetical protein | N/A | N/A | N/A |
| AT1G29230 | *CIPK18* | 2.94 | CBL-interacting protein kinase 18 |  |  |  |
| AT1G29830 |  | 5.95 | magnesium transporter CorA-like family protein | N/A | N/A | N/A |
| AT1G30550 |  | 7.03 | S-adenosyl-L-methionine-dependent methyltransferase superfamily protein |  |  |  |
| AT1G33100 |  | 3.93 | MATE efflux family protein |  |  | 2.52 |
| AT1G33840 |  | 2.47 | LURP-one-like protein (DUF567) |  |  |  |
| AT1G33960 | *AIG1* | 3.06 | P-loop containing nucleoside triphosphate hydrolases superfamily protein | 4.59 | 4.79 | 6.69 |
| AT1G34050 |  | 2.66 | ankyrin repeat family protein | N/A | N/A | N/A |
| AT1G35910 | *TPPD* | 2.11 | haloacid dehalogenase-like hydrolase (HAD) superfamily protein | 11.05 | 34.72 | 4.57 |
| AT1G36060 |  | 2.38 | Integrase-type DNA-binding superfamily protein |  | 2.2 |  |
| AT1G44180 |  | 2.88 | peptidase M20/M25/M40 family protein |  |  |  |
| AT1G44414 |  | 3.28 | zinc-ribbon domain protein |  | 2.15 | 2.39 |
| AT1G49960 |  | 2.54 | xanthine/uracil permease family protein | 2.72 |  | 2.63 |
| AT1G51470 | *BGLU35* | 59.75 | beta glucosidase 35 |  | 2.19 |  |
| AT1G51790 |  | 2.26 | leucine-rich repeat protein kinase family protein |  |  |  |
| AT1G54000 | *GLL22* | 2.79 | GDSL-like Lipase/Acylhydrolase superfamily protein | 4.91 |  |  |
| AT1G57630 |  | 4.06 | Toll-Interleukin-Resistance (TIR) domain family protein | 6.43 | 16.58 | 32.9 |
| AT1G61255 |  | 4.20 | hypothetical protein | N/A | N/A | N/A |
| AT1G61480 |  | 3.29 | S-locus lectin protein kinase family protein | 10.05 | 7.98 | 9.24 |
| AT1G62280 | *SLAH1* | 2.83 | SLAC1 homologue 1 |  | 2.11 |  |
| AT1G64590 |  | 2.24 | NAD(P)-binding Rossmann-fold superfamily protein |  | 2.42 |  |
| AT1G64920 |  | 4.69 | UDP-Glycosyltransferase superfamily protein | 3.92 | 5.77 |  |
| AT1G65730 | *YSL7* | 2.40 | YELLOW STRIPE like 7 | 3.92 | 6.02 | 3.43 |
| AT1G66270 | *BGLU21* | 20.61 | glycosyl hydrolase superfamily protein | 19.88 | 8.64 | 3.31 |
| AT1G66280 | *BGLU22* | 2.69 | glycosyl hydrolase superfamily protein | 19.88 | 8.64 | 3.31 |
| AT1G66465 |  | 2.73 | transmembrane protein | N/A | N/A | N/A |
| AT1G67920 |  | 2.28 | hypothetical protein | 2.44 | 10.94 | 60.04 |
| AT1G68320 | *MYB62* | 5.77 | myb domain protein 62 |  | 2.88 | 2.56 |
| AT1G70460 | *PERK13* | 2.36 | root hair specific 10 |  | 2.13 | 2.17 |
| AT1G70880 |  | 2.92 | polyketide cyclase/dehydrase and lipid transport superfamily protein |  |  |  |
| AT1G72860 |  | 2.46 | disease resistance protein (TIR-NBS-LRR class) family |  |  |  |
| AT1G73340 |  | 2.86 | cytochrome P450 superfamily protein |  | 3.74 | 2.42 |
| AT1G75620 |  | 3.31 | glyoxal oxidase-related protein |  |  |  |
| AT1G77960 |  | 2.94 | repressor ROX1-like protein | N/A | N/A | N/A |
| AT1G79580 | *SMB* | 3.58 | NAC (No Apical Meristem) domain transcriptional regulator superfamily protein | 2.08 |  |  |
| AT2G05240 |  | 2.25 | hypothetical protein | N/A | N/A | N/A |
| AT2G07820 |  | 2.24 | CAP (Cysteine-rich secretory proteins, Antigen 5, and Pathogenesis-related 1 protein) superfamily protein |  |  |  |
| AT2G08185 |  | 2.40 | uncharacterized misc_RNA | N/A | N/A | N/A |
| AT2G09335 |  | 2.21 | other RNA (lncRNA) | N/A | N/A | N/A |
| AT2G15030 |  | 2.49 |  | N/A | N/A | N/A |
| AT2G15370 | *FUT5* | 3.40 | fucosyltransferase 5 |  |  |  |
| AT2G17740 |  | 2.87 | cysteine/histidine-rich C1 domain family protein |  |  |  |
| AT2G18690 |  | 2.23 | transmembrane protein | 3.1 | 5.04 | 29.2 |
| AT2G22510 |  | 4.13 | hydroxyproline-rich glycoprotein family protein | 2.24 | 2.81 | 3.78 |
| AT2G22750 |  | 5.03 | basic helix-loop-helix (bHLH) DNA-binding superfamily protein | 2.81 | 6.0 | 4.05 |
| AT2G22810 | *ACS4* | 2.74 | 1-aminocyclopropane-1-carboxylate synthase 4 | 2.25 | 2.04 |  |
| AT2G25260 |  | 2.80 | Hyp O-arabinosyltransferase-like protein | N/A | N/A | N/A |
| AT2G26410 | *Iqd4* | 3.11 | IQ-domain 4 | 2.17 |  |  |
| AT2G26480 | *UGT76D1* | 3.49 | UDP-glucosyl transferase 76D1 |  | 2.31 |  |
| AT2G27000 | *CYP705A8* | 2.74 | cytochrome P450, family 705, subfamily A, polypeptide 8 | 2.62 | 2.91 |  |
| AT2G29460 | *GSTU4* | 6.25 | glutathione S-transferase tau 4 | 14.72 | 27.34 | 88.56 |
| AT2G30130 | *ASL5* | 2.34 | lateral organ boundaries (LOB) domain family protein | 2.16 | 4.85 | 3.95 |
| AT2G30750 | *CYP71A12* | 4.62 | cytochrome P450 family 71 polypeptide | 21.14 | 9.03 | 6.39 |
| AT2G34350 |  | 2.31 | nodulin-like / Major Facilitator Superfamily protein | N/A | N/A | N/A |
| AT2G35200 |  | 2.38 | DUF740 family protein | N/A | N/A | N/A |
| AT2G35850 |  | 3.29 | transmembrane protein | N/A | N/A | N/A |
| AT2G35980 | *YLS9* | 5.08 | late embryogenesis abundant (LEA) hydroxyproline-rich glycoprotein family | 4.95 | 40.28 | 39.62 |
| AT2G36690 |  | 3.04 | 2-oxoglutarate (2OG) and Fe(II)-dependent oxygenase superfamily protein |  |  | 3.34 |
| AT2G38390 |  | 2.33 | peroxidase superfamily protein | 8.11 | 4.87 | 4.57 |
| AT2G39370 | *MAKR4* | 2.74 | membrane-associated kinase regulator | 2.7 |  |  |
| AT2G40113 |  | 3.06 | Pollen Ole e 1 allergen and extensin family protein | N/A | N/A | N/A |
| AT2G41750 |  | 2.91 | DTW domain-containing protein | N/A | N/A | N/A |
| AT2G43000 | *NAC042* | 3.89 | NAC domain containing protein 42 |  | 4.0 | 5.57 |
| AT2G43470 |  | 2.44 | zinc finger CCCH domain protein, putative (DUF3755) | N/A | N/A | N/A |
| AT2G44000 |  | 3.67 | late embryogenesis abundant (LEA) hydroxyproline-rich glycoprotein family | 2.04 | 3.68 | 2.76 |
| AT2G46950 | *CYP709B2* | 3.39 | cytochrome P450, family 709, subfamily B, polypeptide 2 |  | 2.33 | 7.31 |
| AT2G47540 |  | 7.46 | Pollen Ole e 1 allergen and extensin family protein |  |  |  |
| AT3G01950 |  | 2.45 | peroxidase (DUF 3339) | N/A | N/A | N/A |
| AT3G03500 |  | 5.64 | TatD related DNase | N/A | N/A | N/A |
| AT3G03670 |  | 3.49 | peroxidase superfamily protein | 2.87 |  |  |
| AT3G04105 |  | 3.08 | uncharacterized misc_RNA | N/A | N/A | N/A |
| AT3G04220 |  | 2.84 | disease resistance protein (TIR-NBS-LRR class) family | 4.44 | 8.65 | 2.05 |
| AT3G05155 |  | 4.52 | major facilitator superfamily protein | N/A | N/A | N/A |
| AT3G06505 |  | 3.86 | uncharacterized misc_RNA | N/A | N/A | N/A |
| AT3G07000 |  | 2.77 | cysteine/histidine-rich C1 domain family protein |  |  |  |
| AT3G08595 |  | 2.20 | other RNA (lncRNA) | N/A | N/A | N/A |
| AT3G09940 | *MDHAR* | 3.89 | monodehydroascorbate reductase | 14.76 | 5.8 | 29.92 |
| AT3G11550 | *CASP2* | 2.63 | uncharacterized protein family (UPF0497) |  | 2.78 |  |
| AT3G16390 | *NSP3* | 2.45 | nitrile specifier protein 3 | 4.33 |  | 2.87 |
| AT3G17460 |  | 3.11 | PHD finger family protein | N/A | N/A | N/A |
| AT3G18250 |  | 3.14 | putative membrane lipoprotein | 10.27 | 7.02 | 19.65 |
| AT3G20710 |  | 4.15 | F-box family protein | 2.3 |  |  |
| AT3G21080 |  | 2.25 | ABC transporter-like protein | 3.78 | 6.68 | 4.08 |
| AT3G24210 |  | 3.22 | ankyrin repeat family protein |  |  |  |
| AT3G26125 | *CYP86C2* | 2.82 | cytochrome P450, family 86, subfamily C, polypeptide 2 |  |  |  |
| AT3G26830 | *PAD3* | 5.13 | cytochrome P450 superfamily protein | 3.14 | 13.18 | 11.86 |
| AT3G27270 |  | 2.15 | TRAM, LAG1 and CLN8 (TLC) lipid-sensing domain containing protein | 2.97 | 3.68 | 2.8 |
| AT3G27490 |  | 3.42 | cysteine/histidine-rich C1 domain family protein | 4.64 | 5.79 | 9.58 |
| AT3G27884 |  | 2.63 | other RNA (ncRNA) | N/A | N/A | N/A |
| AT3G29250 | *SDR4* | 3.19 | NAD(P)-binding Rossmann-fold superfamily protein | 2.01 |  | 93.28 |
| AT3G30720 | *QQS* | 6.51 | qua-quine starch |  |  | 3.09 |
| AT3G42800 |  | 2.42 | AF-like protein |  | 2.25 |  |
| AT3G44550 | *FAR5* | 2.81 | fatty acid reductase 5 |  |  | 2.78 |
| AT3G45080 |  | 2.28 | P-loop containing nucleoside triphosphate hydrolases superfamily protein | 2.31 | 2.54 | 2.34 |
| AT3G45280 | *SYP72* | 3.71 | syntaxin of plants 72 | 3.09 | 4.07 | 3.44 |
| AT3G45840 |  | 4.03 | cysteine/histidine-rich C1 domain family protein | N/A | N/A | N/A |
| AT3G46090 | *ZAT7* | 3.04 | C2H2 and C2HC zinc fingers superfamily protein | N/A | N/A | N/A |
| AT3G48201 | *MIR861* | 4.16 | microRNA ath-MIR861 precursor | N/A | N/A | N/A |
| AT3G48400 |  | 4.84 | cysteine/histidine-rich C1 domain family protein | N/A | N/A | N/A |
| AT3G50290 |  | 4.19 | HXXXD-type acyl-transferase family protein | N/A | N/A | N/A |
| AT3G50770 | *CML41* | 3.58 | calmodulin-like 41 |  | 3.49 | 4.25 |
| AT3G51680 | *SDR2* | 3.80 | NAD(P)-binding Rossmann-fold superfamily protein | 2.28 | 7.42 |  |
| AT3G52460 |  | 2.84 | hydroxyproline-rich glycoprotein family protein | 2.26 |  |  |
| AT3G52790 |  | 2.34 | peptidoglycan-binding LysM domain-containing protein | N/A | N/A | N/A |
| AT3G54150 |  | 2.86 | S-adenosyl-L-methionine-dependent methyltransferases superfamily protein | 4.03 | 11.8 | 5.33 |
| AT3G55150 | *EXO70H1* | 3.25 | exocyst subunit exo70 family protein H1 |  | 2.86 |  |
| AT3G55910 |  | 2.72 | ADP-ribosylation factor GTPase-activating protein |  |  | 4.24 |
| AT3G55970 | *JRG21* | 3.85 | jasmonate-regulated gene 21 | 51.84 | 85.77 | 20.38 |
| AT3G58540 |  | 2.92 | hypothetical protein | 2.95 | 2.75 |  |
| AT3G59370 |  | 3.40 | vacuolar calcium-binding protein-like protein |  |  |  |
| AT3G59930 |  | 3.90 | defensin-like protein | 7.15 | 7.75 | 23.62 |
| AT3G62100 | *IAA30* | 2.44 | indole-3-acetic acid inducible 30 |  | 28.27 | 2.76 |
| AT4G00680 | *ADF8* | 3.61 | actin depolymerizing factor 8 | 2.81 | 5.32 |  |
| AT4G02180 |  | 4.58 | DC1 domain-containing protein |  |  |  |
| AT4G04223 |  | 2.03 | other RNA (ncRNA) | N/A | N/A | N/A |
| AT4G04490 | *CRK36* | 2.07 | cysteine-rich RLK (RECEPTOR-like protein kinase) 36 | 9.96 | 178.21 | 40.15 |
| AT4G05100 | *MYB74* | 2.29 | myb domain protein 74 | 3.69 | 10.79 | 16.67 |
| AT4G05200 | *CRK25* | 2.36 | cysteine-rich RLK (RECEPTOR-like protein kinase) 25 |  |  |  |
| AT4G07820 |  | 2.24 | CAP (Cysteine-rich secretory proteins, Antigen 5, and Pathogenesis-related 1 protein) superfamily protein |  |  |  |
| AT4G08450 |  | 10.34 | disease resistance protein (TIR-NBS-LRR class) family | 6.83 | 23.55 | 8.13 |
| AT4G08555 |  | 2.19 | hypothetical protein | N/A | N/A | N/A |
| AT4G08770 | *Prx37* | 2.32 | peroxidase superfamily protein | 4.29 | 4.14 | 40.6 |
| AT4G09415 |  | 3.02 | other RNA (lncRNA) | N/A | N/A | N/A |
| AT4G10500 |  | 2.21 | 2-oxoglutarate (2OG) and Fe(II)-dependent oxygenase superfamily protein | 6.39 | 8.13 | 10.72 |
| AT4G11210 |  | 3.86 | disease resistance-responsive (dirigent-like protein) family protein |  |  |  |
| AT4G12050 |  | 10.11 | putative AT-hook DNA-binding family protein | N/A | N/A | N/A |
| AT4G12510 |  | 6.10 | bifunctional inhibitor/lipid-transfer protein/seed storage 2S albumin superfamily protein |  |  |  |
| AT4G12520 |  | 6.73 | bifunctional inhibitor/lipid-transfer protein/seed storage 2S albumin superfamily protein |  |  |  |
| AT4G13235 | *EDA21* | 2.13 | embryo sac development arrest 21 |  | 2.14 |  |
| AT4G15975 |  | 5.82 | RING/U-box superfamily protein | N/A | N/A | N/A |
| AT4G17483 |  | 2.44 | alpha/beta-Hydrolases superfamily protein |  | 2.6 | 2.5 |
| AT4G17660 |  | 5.03 | protein kinase superfamily protein | 2.12 | 2.9 | 4.5 |
| AT4G18250 |  | 2.23 | receptor Serine/Threonine kinase-like protein | 12.44 | 4.65 | 11.75 |
| AT4G19030 | *NLM1* | 10.65 | NOD26-like major intrinsic protein 1 | 2.02 |  |  |
| AT4G20235 | *CYP71A28* | 2.99 | cytochrome P450, family 71, subfamily A, polypeptide 28 |  |  |  |
| AT4G20460 |  | 2.63 | NAD(P)-binding Rossmann-fold superfamily protein |  |  |  |
| AT4G21850 | *MSRB9* | 2.68 | methionine sulfoxide reductase B9 | 2.71 | 5.12 | 4.95 |
| AT4G22214 |  | 7.36 | defensin-like (DEFL) family protein | N/A | N/A | N/A |
| AT4G22230 |  | 2.70 | defensin-like protein | N/A | N/A | N/A |
| AT4G22610 |  | 2.41 | bifunctional inhibitor/lipid-transfer protein/seed storage 2S albumin superfamily protein | 5.36 | 5.36 | 2.99 |
| AT4G22680 | *MYB85* | 4.53 | myb domain protein 85 | 4.36 | 6.72 | 5.94 |
| AT4G22810 |  | 2.66 | putative AT-hook DNA-binding family protein | N/A | N/A | N/A |
| AT4G24340 |  | 4.91 | phosphorylase superfamily protein | 3.53 | 23.17 | 2.39 |
| AT4G25090 |  | 2.39 | riboflavin synthase-like superfamily protein |  |  |  |
| AT4G25790 |  | 2.88 | CAP (Cysteine-rich secretory proteins, Antigen 5, and Pathogenesis-related 1 protein) superfamily protein |  | 2.2 |  |
| AT4G26560 | *CBL7* | 4.81 | calcineurin B-like protein 7 |  |  |  |
| AT4G28840 |  | 3.51 | mediator of RNA polymerase II transcription subunit | N/A | N/A | N/A |
| AT4G29690 |  | 2.43 | alkaline-phosphatase-like family protein | 3.3 | 3.89 |  |
| AT4G30120 | *HMA3* | 5.53 | heavy metal atpase 3 | 5.72 | 12.0 | 23.2 |
| AT4G30290 | *XTH19* | 2.70 | xyloglucan endotransglucosylase/hydrolase 19 | 6.67 | 5.61 | 3.05 |
| AT4G31110 |  | 2.04 | wall-associated kinase family protein | N/A | N/A | N/A |
| AT4G31320 |  | 3.46 | SAUR-like auxin-responsive protein family |  |  | 2.0 |
| AT4G31875 |  | 3.35 | hypothetical protein |  |  | 2.82 |
| AT4G33560 |  | 4.04 | wound-responsive family protein | 5.2 |  | 2.14 |
| AT4G33720 |  | 4.72 | CAP (Cysteine-rich secretory proteins, Antigen 5, and Pathogenesis-related 1 protein) superfamily protein | 2.03 | 3.62 | 3.02 |
| AT4G33730 |  | 3.17 | CAP (Cysteine-rich secretory proteins, Antigen 5, and Pathogenesis-related 1 protein) superfamily protein |  | 2.65 |  |
| AT4G35200 |  | 2.12 | hypothetical protein (DUF241) | 3.81 |  | 2.32 |
| AT4G36110 | *SAUR9* | 3.15 | SAUR-like auxin-responsive protein family | 2.3 | 4.06 |  |
| AT4G38410 |  | 2.95 | dehydrin family protein |  | 3.19 | 5.43 |
| AT4G38830 | *CRK26* | 2.30 | cysteine-rich RLK (RECEPTOR-like protein kinase) 26 | 2.04 |  |  |
| AT4G39320 |  | 3.20 | microtubule-associated protein-like protein | 2.53 | 3.24 | 2.02 |
| AT5G01165 |  | 2.34 | other RNA (snoRNA) | N/A | N/A | N/A |
| AT5G01490 | *CAX4* | 2.54 | cation exchanger 4 |  | 3.25 |  |
| AT5G02090 |  | 2.05 | hypothetical protein | N/A | N/A | N/A |
| AT5G02350 |  | 4.59 | cysteine/histidine-rich C1 domain family protein | 3.51 | 4.59 | 5.03 |
| AT5G02780 | *GSTL1* | 3.04 | glutathione transferase lambda 1 |  | 3.54 | 19.27 |
| AT5G02865 |  | 2.54 | other RNA (lncRNA) | N/A | N/A | N/A |
| AT5G04120 |  | 7.98 | phosphoglycerate mutase family protein | 4.54 | 8.66 | 7.01 |
| AT5G04190 | *PKS4* | 2.41 | phytochrome kinase substrate 4 | 2.65 | 3.18 | 4.91 |
| AT5G04210 |  | 2.90 | CCCH-type zinc fingerfamily protein with RNA-binding domain-containing protein | 2.02 | 4.04 |  |
| AT5G05250 |  | 2.40 | hypothetical protein | 4.12 | 5.78 |  |
| AT5G05275 |  | 4.04 | other RNA (lncRNA) | N/A | N/A | N/A |
| AT5G05530 |  | 3.17 | RING/U-box superfamily protein | N/A | N/A | N/A |
| AT5G06640 | *EXT10* | 4.10 | proline-rich extensin-like family protein |  | 2.4 |  |
| AT5G08480 |  | 7.69 | VQ motif-containing protein | 2.74 |  | 2.53 |
| AT5G08635 |  | 2.84 | other RNA (lncRNA) | N/A | N/A | N/A |
| AT5G11210 | *GLR2.5* | 3.09 | glutamate receptor 2.5 | 2.0 | 8.41 | 5.69 |
| AT5G12020 | *HSP17.6II* | 5.49 | 17.6 kDa class II heat shock protein | 24.95 | 36.04 | 17.71 |
| AT5G12030 | *HSP17.6A* | 2.70 | heat shock protein 17.6A | 11.71 | 116.73 | 125.45 |
| AT5G13900 |  | 3.04 | bifunctional inhibitor/lipid-transfer protein/seed storage 2S albumin superfamily protein |  |  | 4.06 |
| AT5G13990 | *EXO70C2* | 3.56 | exocyst subunit exo70 family protein C2 |  | 4.41 | 2.1 |
| AT5G14690 |  | 3.15 | transmembrane protein |  |  |  |
| AT5G16170 |  | 2.54 | core-2/I-branching beta-1,6-N-acetylglucosaminyltransferase family protein | N/A | N/A | N/A |
| AT5G17430 | *BBM* | 4.04 | integrase-type DNA-binding superfamily protein |  |  | 3.07 |
| AT5G19700 |  | 2.52 | MATE efflux family protein | 2.15 |  | 4.45 |
| AT5G22788 |  | 2.11 | other RNA (ncRNA) | N/A | N/A | N/A |
| AT5G23990 | *FRO5* | 8.89 | ferric reduction oxidase 5 | N/A | N/A | N/A |
| AT5G24100 |  | 5.05 | leucine-rich repeat protein kinase family protein |  |  |  |
| AT5G24140 | *SQP2* | 2.79 | squalene monooxygenase 2 | 5.69 | 5.5 | 2.87 |
| AT5G24410 | *PGL4* | 2.46 | 6-phosphogluconolactonase 4 |  | 2.59 | 2.58 |
| AT5G24880 |  | 2.51 | chromo domain cec-like protein |  |  | 2.01 |
| AT5G25010 |  | 3.42 | enhanced disease resistance-like protein (DUF1336) | N/A | N/A | N/A |
| AT5G25820 |  | 2.14 | exostosin family protein |  | 2.9 | 9.43 |
| AT5G28080 | *WNK9* | 3.27 | protein kinase superfamily protein |  | 2.13 | 4.52 |
| AT5G33355 |  | 2.75 | defensin-like (DEFL) family protein | 7.1 | 7.75 | 23.62 |
| AT5G38100 |  | 2.29 | S-adenosyl-L-methionine-dependent methyltransferases superfamily protein | N/A | N/A | N/A |
| AT5G38900 |  | 2.88 | thioredoxin superfamily protein | 2.13 | 2.79 | 4.0 |
| AT5G39520 |  | 6.44 | hypothetical protein (DUF1997) |  | 27.67 | 194.66 |
| AT5G40510 |  | 2.79 | sucrase/ferredoxin-like family protein |  |  |  |
| AT5G41590 |  | 2.11 | LURP-one-like protein (DUF567) | N/A | N/A | N/A |
| AT5G43540 |  | 3.50 | C2H2 and C2HC zinc fingers superfamily protein | N/A | N/A | N/A |
| AT5G44460 | *CML43* | 3.40 | calmodulin like 43 |  |  |  |
| AT5G45220 |  | 3.09 | disease resistance protein (TIR-NBS-LRR class) family |  |  |  |
| AT5G46890 |  | 7.35 | bifunctional inhibitor/lipid-transfer protein/seed storage 2S albumin superfamily protein |  |  | 2.29 |
| AT5G46900 |  | 8.93 | bifunctional inhibitor/lipid-transfer protein/seed storage 2S albumin superfamily protein |  |  | 2.29 |
| AT5G47530 |  | 3.58 | auxin-responsive family protein |  | 2.77 | 5.09 |
| AT5G47950 |  | 2.50 | HXXXD-type acyl-transferase family protein |  | 2.09 |  |
| AT5G48000 | *CYP708A2* | 2.30 | cytochrome P450, family 708, subfamily A, polypeptide 2 | 11.43 | 2.38 | 8.3 |
| AT5G49270 | *SHV2* | 4.37 | COBRA-like extracellular glycosyl-phosphatidyl inositol-anchored protein family |  |  |  |
| AT5G50300 | *AZG2* | 3.98 | xanthine/uracil permease family protein |  |  |  |
| AT5G50760 |  | 2.71 | SAUR-like auxin-responsive protein family |  |  |  |
| AT5G51780 |  | 2.46 | basic helix-loop-helix (bHLH) DNA-binding superfamily protein |  |  |  |
| AT5G53250 | *AGP22* | 8.57 | arabinogalactan protein 22 | 2.77 |  | 3.7 |
| AT5G54470 | *BBX29* | 2.78 | B-box type zinc finger family protein | 5.55 | 13.81 | 4.03 |
| AT5G56540 | *AGP14* | 2.76 | arabinogalactan protein 14 |  |  |  |
| AT5G57550 | *XTH25* | 2.33 | xyloglucan endotransglucosylase/hydrolase 25 | 7.54 | 4.92 | 8.35 |
| AT5G57770 |  | 4.03 | auxin canalization protein (DUF828) |  |  |  |
| AT5G59520 | *ZIP2* | 2.19 | ZRT/IRT-like protein 2 |  |  |  |
| AT5G59530 |  | 2.21 | 2-oxoglutarate (2OG) and Fe(II)-dependent oxygenase superfamily protein | 14.67 | 22.78 | 128.3 |
| AT5G60650 |  | 4.07 | proline-rich receptor-like kinase | N/A | N/A | N/A |
| AT5G61260 |  | 2.81 | plant calmodulin-binding protein-like protein | 3.99 | 5.22 | 5.22 |
| AT5G62280 |  | 2.46 | DUF1442 family protein (DUF1442) |  |  |  |
| AT5G62480 | *GSTU9* | 2.46 | glutathione S-transferase tau 9 | 5.79 | 19.57 | 109.89 |
| AT5G66710 |  | 2.27 | protein kinase superfamily protein |  | 2.05 | 2.28 |

^a^ Fold induction was generated from the normalized expression value of each gene in GJW24-treated samples divided by the value in mock-treated samples, based on transcriptome sequencing. The genes whose expression is increased more than twofold in GJW24-treated samples compared to mock-treated samples are listed here.

^b^ Gene description was based on information from NCBI.

^c^ Fold induction was based on information from the Arabidopsis eFP Browser. For each gene, the expression values from drought-treated samples (approximately 10% fresh weight loss) at various time points (0.25, 0.5, 1, 3, 6, 12 or 24 h), 150 mM NaCl-treated samples (salt) at various time points (0.5, 1, 3, 6, 12 or 24 h) or 300 mM mannitol-treated samples (osmotic) at various time points (0.5, 1, 3, 6, 12 or 24 h) were divided by the values from untreated samples at the same time points. The maximum fold increase for each gene was then retrieved. Increases of more than twofold in response to the abiotic stresses are shown in the list.

^d^ ‘N/A (not available)’ means that the expression value of the corresponding gene cannot be retrieved from the datasource of the Arabidopsis eFP Browser
